# Voluntary Associations and Net Fertility During the Swedish Demographic Transition

**DOI:** 10.1007/s10680-018-9465-5

**Published:** 2018-01-15

**Authors:** Johan Junkka

**Affiliations:** Department of Historical, Philosophical and Religious Studies, and Centre for Demographic and Ageing Research, Umeå University, 901 87 Umeå, Sweden

**Keywords:** Fertility transition, Social networks, Voluntary associations, Demographic transition

## Abstract

This study investigates the role of changing social relations for fertility decline during the European fertility transition. The growth of voluntary associations at the end of the nineteenth century entailed a radical shift in the landscape of social relations in Sweden. By combining micro-census data from 1890 to 1900 with local-level membership data for three voluntary association groups, this article assesses the effect of parish-level voluntary association size on net fertility in Sweden using mixed-effects Poisson regression models. The results show that the adoption of fertility limitation during the transition period was associated with the creation and diffusion of the idea of respectability within large social network organisations, an idea that has previously been shown to be connected to fertility limitation. Furthermore, by applying a social network perspective, the results show that the strength of the effect was dependent on the structure of the social networks in terms of size, density, and homogeneity. Voluntary association size had the strongest effect for the free churches, which created dense heterogeneous networks through systems of social control, while the size of the temperance association showed no effect on fertility because the connections between nodes were sparse.

## Introduction

This study investigates the role of changing social relations for fertility decline during the European fertility transition in the late nineteenth century. Studies of post-transitional populations have shown that fertility decisions are always influenced by the social interactions on which they depend. Thus, researchers have focused on how social networks influence actors’ perceptions of reproductive practices and on the role played by social networks in diffusing ideational effects on fertility (Balbo and Barban 2014; Bernardi and Klaerner 2014; Keim 2011; Rossier and Bernardi 2009). Both ideational factors (Coale and Watkins 1986; Lesthaeghe and Surkyn 1988; Lesthaeghe and Wilson 1986) and diffusion mechanisms (Bras 2014; Casterline 2001a; Palloni 2001; Van Bavel 2004; Vanhaute and Matthys 2007; Watkins et al. 1987) are seen as central explanations for the fertility transition; however, social network theory has not been utilised to the same extent. Social network theory provides a middle-range explanation for how ideas and values are connected to individual practices through social network mechanisms (Bernardi and Klaerner 2014).

This study contributes to the field by applying a social network perspective to the analysis of the relationship between voluntary association activity and fertility during the European demographic transition. For this analysis, the voluntary associations provide distinct social networks that were ideationally driven and operated independently of traditional social network structures, such as family or parish communities; thus, they represent new forms of horizontal networks connecting individuals through voluntary interactions. The study presented here was enabled through the use of unique historical data on the size and location of local-level Swedish voluntary association groups in combination with individual-level micro-census data from 1890 and 1900. The aim was to assess the effect of voluntary association size on individual-level fertility during the demographic transition.

In a Swedish context, the growth of voluntary associations at the end of the nineteenth century entailed a radical change in the landscape of social relations; these were mass movements that connected individuals across traditional boundaries and enabled social change (Ambjörnsson 1993; Horgby 1993; Lundkvist 1980). The idea of respectability was an important part of the identity creation process within voluntary associations, and this idea has in turn been shown to be associated with family limitation during the Swedish demographic transition. Respectable families became associated with small families, and birth control became an important practice to conform to these ideals (Horgby 1993; Kling 2007; Skeggs 1997). This suggests that the voluntary associations were important for the creation and diffusion of new ideals about gender, family, and reproduction. The link between respectability and low fertility has been argued to explain free-church members relatively early adoption of fertility limitation during the transition in a region of northern Sweden (Junkka and Edvinsson 2016). Another contribution of this study is thus to test if these patterns can be generalised across a larger population by modelling the possible effects of voluntary association activity on fertility on a national scale.

The objectives of the study were first to identify the social network structures of three voluntary association types—free churches, temperance associations, and workers’ organisations. The second objective was to estimate the size of the three association types at the parish level, and the third objective was to estimate the impact of voluntary association size on the number of children under the age of five on a national scale in 1890 and 1900 while controlling for other factors that are known to affect fertility. The final objective was to estimate how the relationship between voluntary association size and fertility changed over time and between rural and urban environments.

## Theory

The influence of social relations on fertility has been central to the study of diffusion processes (Casterline 2001a; Kohler 2001; Kohler et al. 2001; Watkins 1987, 1990). In sociology, changes in collective behaviours are seen as the result of diffusion processes that work through interpersonal relationships within social networks (Brown 1981; Rogers 2003). Diffusion processes leading to the emergence of collective behaviours are especially strong within formalised networks such as voluntary associations, and the effect of diffusion processes increase as more members join the organisations (Marwell and Oliver 1993). Fertility studies have mainly focused on the diffusion of innovation.

It has been proposed that in pre-transitional populations fertility was only controlled by societal restraints such as marriage patterns or by breastfeeding customs, not by individual choice (Henry 1961). The innovation was the idea of “numeracy of children” aimed at a target family size (Van de Walle 1992) wherein fertility came “within the calculus of conscious choice” (Coale 1976). Through forces such as secularisation and individualisation, birth control decisions became a conscious choice and were thereby transferred from a societal level to that of individual couples (Lesthaeghe and Wilson 1986). However, a number of studies have challenged such a transfer of birth control decisions. Some have shown that birth control was practised by pre-transitional populations and that couples have always consciously used birth control measures to manipulate their fertility outcomes (Bengtsson and Dribe 2006; Kolk 2011; Sandström and Vikström 2015; Van Bavel and Kok 2010). Other studies have shown that in post-transitional populations, fertility decisions have never been fully in the hands of the individual couple, and such decisions have always been made within a context that has influenced different actors’ perceptions of family formation (Balbo and Barban 2014; Morgan et al. 2012; Rossier and Bernardi 2009).

Diffusion of innovation has been contrasted with the process of adjustment by Carlsson (1966). Adjustment refers to a process where couples change their reproductive practices to adapt to new conditions such as the supply of and demand for children (Easterlin and Crimmins 1985). The supply of children would thus be determined by mortality, although such a relationship was questioned by the Princeton European Fertility Project (Coale and Watkins 1986). However, recent studies using disaggregated data have shown the impact of increased child survival rates on reproductive practice both before and during the European fertility transition. High child mortality constrained couples’ agency to manipulate their reproduction, but as mortality rates declined, the space for active manipulation increased, enabling falling fertility rates (Reher and Sandström 2015; Reher et al. 2017; Van Poppel et al. 2012). The demand for children shifted in response to changing perceptions of their cost related to structural changes such as industrialisation and urbanisation. As the modes of production shifted, the incentives for investments in the quality of children through education increased, replacing a preference for the quantity of children (Becker 1960; Guinnane 2011). The effects of these changes were seen first within the upper and middle classes, where the return on investments in child quality would have had its greatest effects (Dribe and Scalone 2014; Dribe et al. 2014).

Social network theory provides an alternative approach to the diffusion of innovation perspective when analysing social change and, in particular, the problem of capturing the relationship between the individual and society. The theory begins from the perspective on human interactions that “social actors are dependent on their structural environment; i.e. on society, on an organisation, or on the networks of personal relations in which they are embedded”. Yet, “this structure does not fully determine their actions” and thereby allows for individual agency (Bernardi and Klaerner 2014: 644). The structural environment builds the context of the action; it limits, enables, and shapes actors’ interests and perceptions (Burt 1982: 9; Keim 2011: 24–25). Thus, from the perspective of fertility studies, social network theory provides hypothetical links between social organisations and reproductive practices through social interaction (Bernardi and Klaerner 2014: 644).

The mechanisms through which social interaction can lead to social change are manifold, two of which are relevant for the study of fertility and voluntary associations. First, reproductive practices can be diffused through social learning (Casterline 2001b). After observing the behaviours of others and evaluating the consequences of such behaviour, individuals will adopt or reject certain practices (Bernardi and Klaerner 2014: 645). Social learning is argued to have been important for the diffusion of birth control between social classes during the Belgian fertility decline of the late nineteenth century (Van Bavel 2004) and in other studies where domestic servants are argued to have adopted their employers’ norms and behaviours regarding reproductive practices, thus diffusing ideas and practices that valued family limitation (Vanhaute and Matthys 2007).

Second, reproductive practices can be influenced through social pressure. This is the mechanism that encourages individuals to conform to social norms and to the expectations of others in order to gain approval or avoid sanctions. Both implicit and explicit cultural or institutional norms can shape the way individuals perceive the meaning of reproductive practices and how they change their behaviours accordingly (Bernardi and Klaerner 2014: 645). Studies of demographic transitions that have incorporated cultural, political, and ideological factors in their analyses of fertility have shown that social pressure mechanisms have an impact on fertility outcomes (Hacker 1999; Lesthaeghe and Surkyn 1988; Van Poppel and Derosas 2006).

The impact of social organisations also depends on the structure of the network. Not only could its ideologies, norms, and values affect reproductive practices, but so could its size, density, and homogeneity. Size refers to the number of nodes (individuals) within the network, and density refers to the number and strength of the connections between nodes. The homogeneity of the network refers to similarities between network partners (Keim 2011: 22). This has also been argued to be a separate mechanism of social contagion, in which a person adopts an idea or behaviour from another person because of social similarity (Bernardi and Klaerner 2014: 645). The more similar the nodes are within a network, for example, in age, the stronger the mechanism. Studies on the formation of social networks support this hypothesis. During the early twentieth-century individuals were more likely to join a temperance association the more socially similar the individuals were to the existing members (Marsden and Friedkin 1994; Sandell and Stern 1998). This has also been seen in studies on the Dutch fertility transition where individuals with high age-homogeneous networks had fewer children than those with diverse networks such as baptism witnesses (Bras 2014).

## Swedish Voluntary Associations

A voluntary association in this study refers to an organisation that falls within the term “popular movement”, or Folkrörelsen in Swedish. Such an association (1) “arises in order to promote some common interest of its members”; (2) where “membership is voluntary”; and (3) “the organisation exists independently of the state” (Lundkvist 1980: 226). Other criteria are that (4) the members work for the organisation in their spare time (Sills 1968) and (5) that the organisation has a large membership (Lundkvist 1980: 226). Within this definition there are three types of Swedish voluntary associations: the free church congregations, the temperance organisations, and the workers’ organisations such as trade unions and the socialist political party. The definition of voluntary association excludes organisations such as the bourgeois women’s movement because their membership numbers were relatively small and were thus not a mass movement on the same scale as the other three types of associations. Although they had a strong stance on family issues (Gordon 1973; Kling 2007), due to their small numbers their impact cannot be captured in a similar way to the other organisations.

**Fig. 1:**
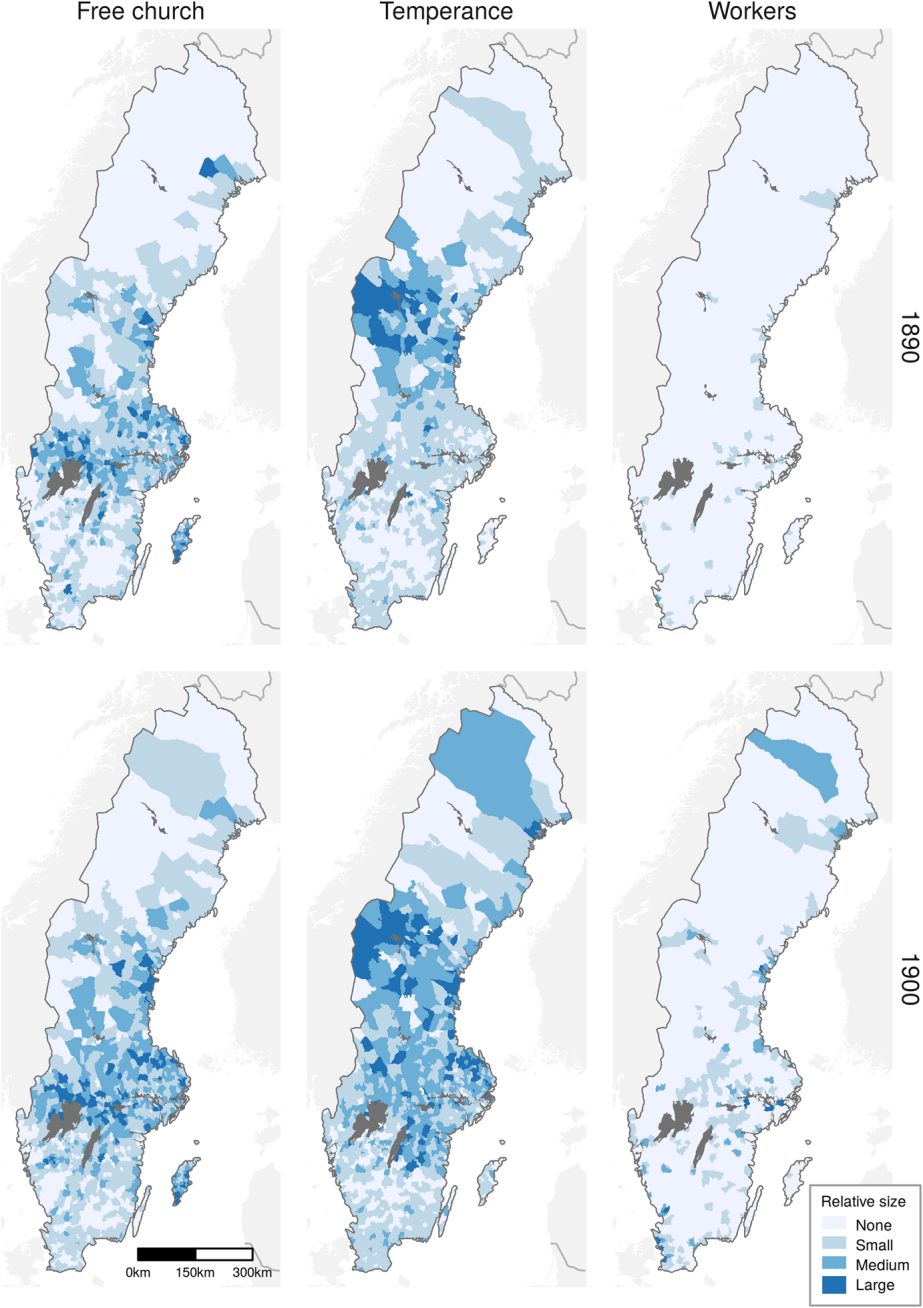
Spatial distribution of voluntary associations by relative size in 1890 and 1900. Sources: Statistics Sweden (2015) and Hofsten and Lundström (1976)

The growth of voluntary associations during the nineteenth and early twentieth-centuries has been linked to industrialisation, and Anderson (1971) sees the associations as a stabilising factor during industrialisation by providing mechanisms for traditional cultural and social hierarchies to adapt and modernise. British friendly societies in the early nineteenth century have been linked to migration and changing occupational structure; however, the friendly societies mainly provided mutual aid to vulnerable young migrants and did not display forms of organisational structure similar to the Swedish voluntary associations (Gorsky 1998). The emergence of the Swedish voluntary associations has been similarly linked to urbanisation and industrialisation, or rather to the class conflict that such changes resulted in. The associations gathered disaffected individuals and were thus important in the struggle for power at that time (Lundkvist 1980). However, the link between voluntary associations and industrialisation has been contested. American voluntary association growth has been shown to be independent of immigration, industrialisation, and urbanisation. The American associations were stronger in rural areas and small towns than in densely populated areas, and Gamm and Putnam (1999) hypothesise that it was easier for them to establish themselves in small homogenous communities where everyone knew each other than in heterogeneous urban areas. Studies of post-transitional societies have not found any evidence for the link between voluntary association growth and industrialisation; instead, in a national comparative analysis, the size of such associations was shown to be strongly correlated with population age structure and education (Curtis et al. 1992).

In Sweden, fertility started to decline around the 1880s, reaching its lowest point in the 1930s when fertility rates were less than 2 children per women, a decline from 4.2 children before the transition. At the same time as fertility declined, Swedish voluntary associations steadily grew in numbers from 1880, with different groups having quite different trajectories. After a period of rapid growth in the 1880s, the free churches stagnated with a membership at around 5% of the adult population. The temperance movement reached its peak around 1910, and then steadily shrank into the 1930s. The trade unions and political organisations displayed constant growth from the 1890s onward, encompassing a quarter of the adult population in the 1940s (Lundkvist 1977).

Even though most areas of Sweden followed the same pattern, there were large spatial variations in the spread and size of the different voluntary association types as seen in Fig. 1. Most notable is the strong presence of the temperance associations and free churches in rural areas and the concentration of the workers’ movement in urban areas, especially in 1890 when the movement was in its infancy.

**Fig. 2:**
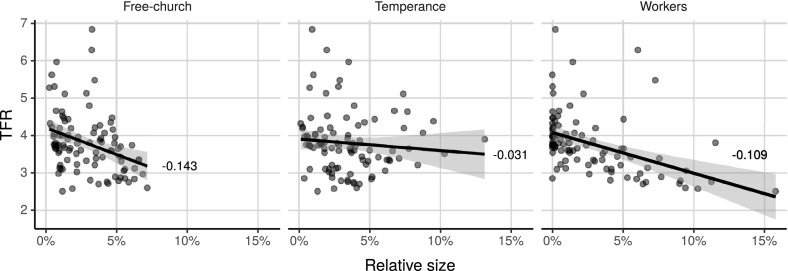
Relationship between county-level voluntary movement size and total fertility rate (TFR) 1890–1920. Sources: Andrae and Lundkvist (1998), Hofsten and Lundström (1976) and Statistics Sweden (2015)

The association with voluntary association growth is also seen at the county level. Using total fertility rate data at the county level for every tenth year (Hofsten and Lundström 1976) in combination with county-level relative voluntary association size between 1890 and 1920 (Andrae and Lundkvist 1998; Statistics Sweden 2015), the correlations can be explored further. Each point in Fig. 2 represents the observed total fertility rate and the size of the voluntary association group as a percentage of the adult population in a county at 1 year. As seen by the fitted line in the scatter plots, there was a negative correlation between the size of voluntary associations and total fertility rate during this period. The negative correlation was stronger for the free churches than the workers’ organisations, while there seems to have been no significant correlation for the temperance organisations.

Although the observations indicate a strong relationship dependent on both the structure and size of the voluntary association types, these are only correlations and do not show causation. The effects could also be caused by structural differences between the counties or by compositional differences in the voluntary association members. This highlights the importance of analysing the effect at an individual level where regression models make it possible to control for individual attributes such as age and occupation as well as contextual-level factors affecting all individuals within a certain area, such as the level of industrialisation and urbanisation, both of which contribute to geographical heterogeneity in fertility.

### Ideational Factors

Lesthaeghe and Surkyn (1988) identified three central ideational factors for changing fertility behaviours during demographic transitions—individualisation, secularisation, and the spread of bourgeois family ideals. The two latter factors were also important ideational attributes of the voluntary associations and were in turn connected to the idea of respectability within the associations.

Secularisation is seen as a process whereby the meaning of religion was challenged and changed resulting in competing interpretations, such as deism and rationality, as well as new forms of Christian associations (McLeod 2000). The voluntary associations in general, and the free churches specifically, were thus expressions of secularisation and represented new ways of conceptualising sexuality and family roles in relation to morality. Within the free churches, sexual restraint and abstinence became connected to female respectability (Brown 2009, Pasture and Art 2012) making small families not only conceivable but also desirable. Men were seen as more easily corrupted by sin; however, it was the wife’s responsibility to lift her husband to the same level of morality as her. In the free churches, masculinity was achieved through the roles of respectable fathers, husbands, and workers and by conforming to ideals of chastity and restraint (Hammar 1999; Pasture and Art 2012). Similarly, respectability has been shown to be important for masculinity and family limitation among men in Lutheran cantons in Switzerland during the historical fertility transition (Praz 2007, 2009). Although the use of contraceptives was not condoned within the free churches, family limitation through abstinence was promoted over alternatives (Hammar 1999; Kristenson 1997). On the issue of birth control, the free churches and the bourgeois women’s movement were thus united in the strive for sexual morality and restraint, while the socialist women’s organisations strove to take active control of their reproduction without similar forms of restraint (Kling 2007).

Embourgeoisement, referring to the adoption of middle-class family ideals by the lower classes, places the attention on the quality of family life (Lesthaeghe and Surkyn 1988). According to Skeggs (1997), respectability becomes a way of positioning oneself as a man or a woman within a certain class, and respectability was most important for those where it was not a given—the working class—and thus was an important part of the embourgeoisement process. This process was, in turn, important for the creation of shared identities within the temperance associations and the workers’ movement. Family ideals were part of the norms that were professed, debated, and formed among their members, who were seen as model citizens embodying the ideals of the association (Ambjörnsson 1993, 1998). These ideals were upheld by differentiating between the respectable and unacceptable through their daily practices, including their reproductive behaviours (Skeggs 1997). Through qualitative studies, Kling (2007) has shown the importance of the idea of respectability for the adoption of birth control during the fertility transition in Sweden. Small families became associated with respectable families, and birth control was an important practice in conforming to these ideals. Birth control was thus not only an aspect of embourgeoisement but was also a part of the identity-creating process within the Swedish voluntary associations and was an important tool for achieving respectability.

### Social Network Structures

As social network organisations, the Swedish voluntary associations were quite homogeneous, gathering young individuals from the working and middle classes. The free churches differed. Although they mainly attracted individuals from the younger age groups, their members came from all social classes. They also deviated in terms of diversity. In contrast to the other movements, the free church members were primarily women, and the churches did not have the same membership turnover as the other organisations. Furthermore, the initiation procedure was more intricate and ritualised than in other organisations, and this made them more stable and able to create dense networks that connected individuals across class boundaries through a common faith (Lundkvist 1977). Because of the importance set on morality and the strong social pressure to conform to acceptable norms (Kennerberg 1996) the mechanisms for social learning and social pressure were strong. Junkka and Edvinsson (2016) found that the free church affiliates in the Sundsvall region had a relatively early decrease in fertility. As ideas about family limitation, respectability, and self-control became integrated into their gendered religious identities, the members used spacing of births through abstinence to conform to social pressures. In addition, aggregate-level studies using crude fertility proxies and qualitative indicators for the strength of free churches have shown connections between low fertility and regions with a strong free church presence and between high fertility and regions with a strong Lutheran state church (Larsson 1984; Lockridge 1983).

The temperance organisations were more homogeneous than the free churches when it came to age and social class, gathering young individuals from the middle class and the skilled working class (Lundkvist 1977). Morality and restraint was the central issue for this movement, so it is easy to deduce that this might have affected their perceptions of reproductive practices. On the other hand, the temperance organisations did not create as dense connections between their members as the free churches because of their membership turnover, which was the highest among the voluntary associations (Lundkvist 1977: 94–97).

The workers’ movement, the unions, and the political organisations were the most homogeneous of the networks, consisting mostly of young working-class men. Issues of morality and restraint were not as central to their agenda, but in certain settings workers’ organisations have been shown to affect fertility. (Warg 2002) linked the adoption of family limitation within the working classes to ideals about manliness and respectability within local political voluntary association organisations and to the diffusion of ideals and practices through the women’s organisations. On the other hand, the early workers’ movement was closely connected to old hierarchical structures, essentially extensions of the existing protectionist craftsmen guilds. It was not until the turn of the century that the movement began to represent a new form of social organisation through the manifestation of the socialist political party (Lundkvist 1977).

## Hypothesis

According to social network theory, which proposes that reproductive practices have always been performed within social networks and have always been affected by both the norms and the structure of the network (Bernardi and Klaerner 2014; Burt 1982; Keim 2011), voluntary associations could shape actors’ interests and their perceptions of family formation. Previous research using individual-level data has shown a relationship between free church affiliation and reproductive control (Junkka and Edvinsson 2016). If such findings are true, we would expect that voluntary associations had a negative effect on fertility and that the strength of the effect was dependent on the social network structure of the voluntary association types in terms of size, density, and homogeneity.

## Data

Data on fertility were obtained from the 1890 and 1900 Swedish censuses. Data were retrieved from the North Atlantic Population Project (NAPP), which has pre-processed and standardised the censuses for research purposes. The censuses provide snapshots of the entire population at a given time, allowing for analysis of fertility using individual-level data. However, the censuses have some limitations. There is no information on all children born, so the numbers observed in the censuses only represent achieved fertility and are biased by differences in child mortality. Mother–child relations are obtained through information on their relationship to the household head. Although this type of relational linking is prone to errors in complex households, with children mismatched to stepmothers or grandmothers, the Swedish censuses have an explicit link between children to their own mothers, which minimises the risk of mismatching (Scalone and Dribe 2017). The procedure is documented on the NAPP website (Minnesota Population Center 2015), and general issues concerning the micro-data have been thoroughly discussed in previous studies (Ruggles et al. 2011; Sobek et al. 2011) as well as specific issues related to the Swedish sample (Scalone and Dribe 2017).

From the censuses, it was impossible to calculate any standard fertility measures, so we were limited to using the observed number of children living with a mother within a household. Because the Swedish fertility transition can be mainly attributed to a decline in marital fertility rather than changes in nuptiality, the analysis focuses on married women (Carlsson 1966; Coale and Watkins 1986). Thus, fertility is measured as the number of children 0–4 years old living within a married woman’s household; thus, the measurement represents fertility for the previous 5-year net mortality. As noted above, net fertility is therefore biased by differences in infant mortality. However, infant mortality was mainly spatially clustered, with large geographical variations during the nineteenth century, especially between urban and rural areas, and has been shown to not display any significant social patterns (Edvinsson 2004; Edvinsson et al. 2008). Scalone and Dribe (2017) have, for example, shown (using the child–woman ratio) that the relative socio-economic differences in fertility calculated from the censuses remained when adjusting for mortality; only the levels were affected. Thus, the main effect of infant mortality on fertility differentials is shared at a local level, contributing to spatially structured geographical heterogeneity, and can be controlled for in a regression model.

To draw conclusions on causality between voluntary association membership and fertility on a national scale, one would need information on association affiliation at the individual level. However, national-level data on voluntary associations are scarce for this period, and individual-level information for the whole population does not exist, so we were limited to using aggregate-level data. Furthermore, previous studies that have shown household-level causal links using longitudinal data have been limited in scope (Junkka and Edvinsson 2016). The use of aggregate data enables an analysis of the association on a national scale, trading the validity of causality for generalisability, which has not been previously studied.

Voluntary association size was estimated using organisational-level membership data collected by the Popular Movement Project (Andrae and Lundkvist 1998). The project’s database has annual membership numbers for almost all local branches of the free churches, temperance associations, and workers’ organisations between 1881 and 1950 (Andrae 1984). The database includes local branches of all large sub-groups belonging to the three organisations, but not smaller sub-groups or youth organisations, which means it only has numbers for adult members (Lundkvist 1977: 62–63). This investigation utilises data from 1885 to 1900 and includes 8121 unique local groups, described further in Table 1. Each observed membership in a local group represents members in the local chapter of the organisation, which had their own board and regular meetings with the local members (Kennerberg 1996; Lundkvist 1977).

**Table 1:** Description of voluntary association data 1885–1900. Source: Andrae and Lundkvist (1998)

	Free church	Temperance associations	Workers’ organisations	Total
Unique local groups	1975	5346	800	8121
Median membership	50	44	30	45
Mean membership	91	60	123	77
Total membership 1890	109,673	62,351	2357	174,381
Total membership 1900	122,567	146,509	83,909	352,985

Although the data capture the absolute majority of the members, estimating the relative size of local groups is not without its problems. Because one individual could belong to multiple organisations, the numbers cannot be taken as the real proportion of the population. However, the multiple registration problem is restricted mostly to the temperance movement, which means that the size of these organisations is overestimated in the data. Another problem is membership turnover, which was large for some organisations within the temperance and workers’ movements (Lundkvist 1977: 94–97). Thus, the data underestimate the number of individuals who came into contact with the movements in some form or other. This highlights the importance of estimating size over a longer period in order to capture trends rather than short-term fluctuations. In addition, membership was not recorded annually for all organisations, but because start dates and end dates of the organisations’ existence are coded in the dataset, it is possible to impute the missing values throughout the lifetime of the organisations.

Yet another problem is that because these organisations were independent of the state, and membership recruitment was not limited by administrative borders, i.e. to a parish. Some organisations, mainly in the workers’ movements, attracted members from large areas outside parish boundaries, occasionally resulting in memberships that outnumbered the adult parish populations. This means that when estimating voluntary association size in relation to parish population, we had to also consider the organisations surrounding a parish.

## Estimating Voluntary Association Size

In this study, the size of the organisation was calculated by parishes, the smallest geographical unit in the censuses. Due to changes in administrative boundaries, the smallest common parish boundaries between 1890 and 1900 were used. Because the organisations attracted members from other parishes, it was not sufficient to aggregate membership numbers by parish. The calculations had to include organisations surrounding each parish. To enable spatial aggregation by parishes, each organisation was geocoded using the Google Geocoding API (Google Developers 2015). The results were controlled by comparing the location of each unique place against the municipal boundaries of 1985, which showed a less than 1% error rate. These errors were then manually geocoded.

Approximations were also made to compensate for the fact that voluntary association membership was not recorded annually or consistently and for the possibility that an organisation had several start and end dates. The missing values were imputed using linear interpolation for irregular time series. This procedure enables imputation for the full lifetime of the organisation, while still allowing for years without members if the organisation had temporarily ceased operations.

In addition, the absolute number of members could not be used because some members might have come from the surrounding area. Therefore, the membership number for each movement was weighted by the population that might belong to that movement. The size of voluntary association type *A* for parish *j* can be estimated as:

1 $$\begin{aligned} A_j = \sum _{d(j,k)<5\,\text {km}} \frac{M_k}{\sum _{d(k,j)<5\,\text {km}}P_j} \end{aligned}$$

where ${M_k}$ is the 5-year averaged number of members in voluntary association *k*, *d*(*j*, *k*) is the distance between parish *j* and voluntary association $k, {P_j}$ is the 5-year averaged adult population in parish *j*, and *d*(*k*, *j*) is the distance between voluntary association *k* and parish *j*. Thus, $\sum _{d(j,k)}$ is the sum of all voluntary associations’ relative memberships within 5 km of the parish, which is given by dividing $M_k$ by $\sum _{d(k,j)<5\,\text {km}}{P_j}$, which is the sum of the 5-year averaged adult population in the parishes within 5 km of voluntary association *k*.

The parish-level voluntary association sizes were divided into four groups for each association type. The first consisted of parishes without a voluntary association presence, and the other three groups were divided into quantiles. The resulting groups were none, small, medium, and large; none was used as the reference.

## Regression Model

Each value of observed fertility was assumed to be determined by a probability distribution. Counting processes with low frequencies such as the number of children under 5 years of age often display a pattern similar to a Poisson distribution. The observed counts fit equally with the theoretical count from a Poisson distribution as with the theoretical count from alternatives such as a negative binomial distribution, and they fit better than the expected values of a zero-inflated negative binomial distribution. Furthermore, it is assumed that the conditional mean of the observed fertility is dependent on both a structural element and an idiosyncratic element. The structural element is the outcome of a number of fixed effects, such as the age of the women and parish-level random effects arising from geographical heterogeneity. The model can be expressed as

2 $$\begin{aligned} \log (\mu ) = X\beta + Zb \end{aligned}$$

where the linear predictor $\beta$ is a matrix of fixed-effects coefficients, *b* is a matrix of random effects, and *X* and *Z* are the model matrices used to determine the conditional mean $\mu$ given the random effects of *b* through the log link function (Bates 2010: 83–85).

In addition to voluntary association size, the fixed effects are parish-level urbanisation and census year and adjustment factors affecting the perceived supply and demand of children, which are the age of the wife, socio-economic status classified according to the HISCLASS 5 social class structure (Leeuwen and Maas 2011; Leeuwen et al. 2002; Mandemakers et al. 2013), the number of teachers per child as a proxy for investments in child education, the wife’s workforce participation, and the 5-year averaged county-level infant mortality rate centred around the mean as an indicator of the supply of children. By controlling for socio-economic status through the occupational title of the husband, it is possible to differentiate the effects of rural-based occupations such as farmers from the effect of other occupations that are more connected to industrial work, such as skilled workers. Furthermore, because the likelihood of joining a voluntary association was dependent primarily on age and occupational status (Lundkvist 1977; Sandell and Stern 1998), controlling for these factors reduces the risk of biasing the result due to selection effects. The fixed effects are described further in Table 2.

**Table 2:** Descriptive statistics of fixed-effects variables. Source: Minnesota Population Center (2015) and Andrae and Lundkvist (1998)

	Fixed effect	Mean net fertility	Mean
Free church	None (Ref.)	1.05	0.21
	Small	1.00	0.26
	Medium	0.95	0.26
	Large	0.94	0.26
Temperance association	None (Ref.)	1.04	0.17
	Small	0.98	0.28
	Medium	0.95	0.28
	Large	0.97	0.28
Workers’ movement	None (Ref.)	1.01	0.64
	Small	0.99	0.12
	Medium	0.94	0.12
	Large	0.84	0.12
Wife’s age	20–24	1.02	0.07
	25–29 (Ref.)	1.33	0.16
	30–34	1.32	0.19
	34–39	1.15	0.20
	40–44	0.81	0.19
	45–49	0.29	0.17
Wife’s occupation	Not in workforce (Ref.)	0.99	0.99
	In workforce	0.60	0.01
Husband’s HISCLASS	Skilled workers (Ref.)	0.98	0.24
	Elite	0.74	0.02
	Lower middle class	0.86	0.09
	Farmer	1.04	0.23
	Unknown	0.85	0.09
	Unskilled workers	1.03	0.34
Teachers per child	< 0.019 (Ref.)	1.06	0.25
	$\ge$ 0.019–< 0.027	1.02	0.25
	$\ge$ 0.027–< 0.038	0.97	0.25
	$\ge$ 0.038–< 0.500	0.88	0.25
Infant mortality rate			100.87
Urbanisation	Rural (Ref.)	1.01	0.76
	Urban	0.88	0.24
Year	1890 (Ref.)	1.00	0.49
	1900	0.96	0.51

The level of urbanisation was defined using the population density. The parishes were divided into an urban group and a rural group, where a parish with a density greater than 300 persons/km^2^ was considered urban. The definition of urbanisation could have been based on information from official registers; however, because of rapid urbanisation around the turn of the century, the official definition was not sufficiently updated. Thus, the population density parameter functions as a better indicator of urbanisation at that time. Because there was a correlation between urbanisation and industrialisation at this time, the variable also functions as a crude proxy of industrialisation.

Geographical heterogeneity occurs because of structural differences between the parishes such as differences in the level of industrialisation of level of infant mortality. Such heterogeneity could affect both fertility and the size of the voluntary associations. The random effect allows the group-level intercept to vary by parish and thus captures the variance in net fertility explained by structural differences between parishes. Because the interest of this study is only to control for these effects while drawing conclusions for the whole population, and not to draw conclusions about the regional variations, the parish-specific intercepts are treated as random, drawn from a probability distribution that takes the following form:

3 $$\begin{aligned} b\sim N(0,\sigma ^2) \end{aligned}$$

where *b* is a normal probability distribution with a mean of zero and a variance of ${\sigma ^2}$, which is the variance of the parish-specific random effects. The characteristics of the geographical units, the parishes, are shown in Table 3. Whenever possible, the parishes are linked across censuses; however, due to changes in administrative boundaries the total number of unique units is larger than the census units. The regression models were constructed and evaluated using the R (R Core Team 2017) package lme4 (Bates et al. 2014, 2015).

**Table 3:** Description of random effect units Source: Minnesota Population Center (2015) and Andrae and Lundkvist (1998)

	1890	1900
Number of unique units	2466	2469
Mean total population	3927	4207
Mean $\hbox {km}^2$	178	178

## Results

The results of the Poisson regression are shown in Table 4 as incidence rate ratios (IRRs) and standard errors for the 1890 and 1900 censuses. The IRRs are essentially exponentiated coefficients and represent relative differences in mean fertility compared to the reference group. Because of the large sample size, almost all variables have significant effects on net fertility, although the size of the effect shows large variations. In line with previous studies, the results show large fertility differentials by socio-economic status (Dribe and Scalone 2014; Dribe et al. 2014). Even greater is the effect of female workforce participation. Although the number of women registered as part of the workforce was very low, the differences in fertility are very high at 30%. Furthermore, the results show that the more teachers per child within a parish, the lower the net fertility, suggesting that increased investments in child quality were important for declining fertility. However, the education parameter only shows fertility differentials of at most 5%.

**Table 4:** Effects of voluntary association size on the number of children under 5 years in 1890 and 1900 in Sweden. Effects are shown as incident rate ratios (IRRs) and standard errors (SE) from a Poisson regression. Source: Minnesota Population Center (2015) and Andrae and Lundkvist (1998)

	Variable	IRR	SE	*P* value
*Intercept*		1.42	0.006	0.00
Free church	None (Ref.)	1.00		
	Small	0.97	0.005	0.00
	Medium	0.95	0.005	0.00
	Large	0.93	0.006	0.00
Temperance association	None (Ref.)	1.00		
	Small	1.01	0.004	0.10
	Medium	0.99	0.005	0.06
	Large	0.99	0.006	0.14
Workers’ movement	None (Ref.)	1.00		
	Small	1.00	0.005	0.76
	Medium	1.01	0.006	0.03
	Large	0.92	0.007	0.00
Wife’s age	20–24	0.77	0.004	0.00
	25–29 (Ref.)	1.00		
	30–34	1.00	0.003	0.12
	34–39	0.87	0.003	0.00
	40–44	0.61	0.003	0.00
	45–49	0.21	0.005	0.00
Wife’s occupation	Not in workforce (Ref.)	1.00		
	In workforce	0.70	0.012	0.00
Husband’s HISCLASS	Skilled workers (Ref.)	1.00		
	Elite	0.82	0.009	0.00
	Lower middle class	0.93	0.004	0.00
	Farmer	1.07	0.003	0.00
	Unknown	0.87	0.004	0.00
	Unskilled workers	1.04	0.003	0.00
Teachers per child	< 0.019 (Ref.)	1.00		
	$\ge$ 0.019–< 0.027	0.98	0.004	0.00
	$\ge$ 0.027–< 0.038	0.97	0.004	0.00
	$\ge$ 0.038–< 0.500	0.94	0.005	0.00
Infant mortality rate		0.89	0.011	0.00
Urbanisation	Rural (Ref.)	1.00		
	Urban	0.95	0.010	0.00
Year	1890 (Ref.)	1.00		
	1900	0.99	0.003	0.00
Summary	Observations		1,068,224	
	Random effect groups		2521	
	Degrees of freedom		28	
	Standard deviation of			
	parish random effects		0.102	
	Deviance residuals		961,590	
	Residual degrees of freedom		1,068,196	

As expected, the relationship between voluntary associations and net fertility is negative when controlling for census year, urbanisation, and geographical heterogeneity. However, the effects only show significant trends for the size of free churches and workers’ organisations. The differences are at the most 7.5% between populations in parishes with no voluntary associations and those with large voluntary associations. This shows that in parishes where the free churches or the workers’ organisations were strong, more individuals controlled their reproduction to a greater extent.

This does not in itself mean there is a causal link between voluntary associations activity and reproductive practices. People who had started to reduce their fertility might also have been more attracted to the voluntary associations. Having fewer children would make it easier to join an association, especially for women, and the effects could therefore occur due to reversed causation. Without individual-level data on the timing of both births and the start of membership, it is impossible to determine the direction of the effect. However, the results support previous research that connects voluntary association affiliation with family limitation (Junkka and Edvinsson 2016; Larsson 1984; Lockridge 1983) by showing that the relationship is not only confined to a subsection of the population, but can also be generalised across the whole nation.

An alternative explanation for the relationship between voluntary association size and fertility decline is the existence of certain social groups acting as forerunners in changing reproductive behaviours and in joining voluntary association groups. Similar effects have been shown in the Belgian fertility decline, where there were significant spatial diffusion effects at a neighbourhood level that were dependent on both linguistic and occupational structures (Van Bavel 2004). Although the voluntary associations were linguistically homogenous and, in general, also showed a similar occupational structure, there were variations between organisations. This latent variable was not controlled for in the present model because there is no information on the social composition of each local voluntary association group.

**Fig. 3:**
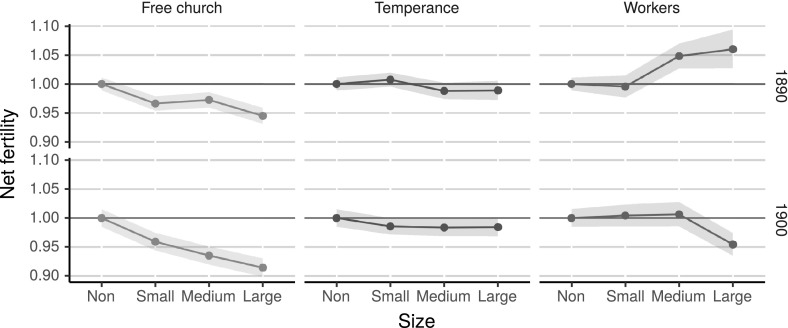
Relative predicted number of children under 5 years of age by voluntary association size in 1890 and 1900 in Sweden based on the Poisson regression shown in Table 5 in “Appendix”

To further test the effect of voluntary association size, we introduced an interaction with census year to capture how the effect changed over time. Figure 3 shows differences in the predicted mean number of children under 5 years of age by voluntary association size in 1890 and 1900. In addition, the figure shows the confidence intervals for the predicted means, which, in contrast to prediction intervals, are narrower because they only apply to the mean and not to single new events (Kerns 2010: 237). The full results of the regression are shown in Table 5.

There were large variations in the effect of voluntary association size on net fertility between groups. When interacting size with census year, we see that the negative effect of the free churches increases over time because the slope of the line increases. This indicates an increase in the difference between parishes with no free churches and those with strong free churches, from 6% in 1890 to almost 10% in 1900. Although there is a significant effect of the medium-sized temperance organisations in 1900, the effect is very small and there is no clear trend in the predicted mean. The largest change is seen in the effect of the workers’ movement, where in 1890 the effect on net fertility is positive, although only significant at a medium-size presence, while the effect was negative in 1900, showing a 6% difference between parishes with no workers’ organisations and those with large ones.

The results show that the negative effect of the free churches and the workers’ organisations on net fertility increased between 1890 and 1900; thus, the effect became stronger as more people were exposed to these networks. The workers’ movement was in its infancy between 1884 and 1889, which is shown by the larger confidence intervals because the number of groups was smaller than for the other association types. The early workers’ movement mainly consisted of craftsmen acting within the traditional hierarchical structure (Lundkvist 1977) and thus represented an old social network structure that was different from the workers’ movement of 1900.

**Fig. 4:**
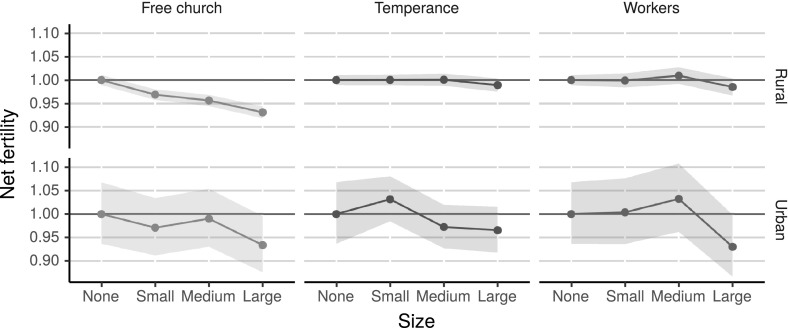
Relative predicted number of children under 5 years of age by voluntary association size in urban and rural parishes in Sweden from the Poisson regression shown in Table 6 in “Appendix”

As suggested by the spatial distribution of the associations, there are strong correlations between urbanisation and the workers’ movement. At that time, the urban population was small, and because these organisations were concentrated in urban areas, the effects of their size are not visible in the previous regression models. Because the workers’ movement would grow to encompass a quarter of the adult population, it is of interest to assess the effect of these organisations as early as possible. Differences in the relationship between voluntary association size and net fertility were explored further by introducing an interaction with urbanisation to the regression model. In addition, because the urban population was small, the censuses were combined to increase model robustness. Figure 4 shows the differences in predicted mean net fertility for different types of voluntary association sizes by context, and the full regression results are shown in Table 6.

Only the free churches had a negative effect on net fertility within rural environments, and, although the confidence intervals are wider within the urban context, the effect is also negative, showing almost the same difference in the predicted mean (around 7%) between parishes with no free churches and those with large ones. The temperance movement had no significant effect in either rural or urban parishes, similar to the results in the previous regression models. For the workers’ movement, it is not clear whether the negative effect was present only in urban environments, and at most it shows a 7% difference in net fertility. Urbanisation can also be seen as a crude proxy for industrialisation because the two processes were often correlated during the time period studied here. The results can therefore be interpreted as showing how the effect of voluntary association size was modified by industrialisation and that the effect of the free churches existed independently of industrialisation, showing clear negative effects on fertility in rural environments.

It is evident that the different effects of free churches and workers’ organisations were not only due to the organisations’ network structures, but also to the structural environment of the context in which they operated. The effects of voluntary association size in urban areas were nonlinear, suggesting a cut-off point. The proportion of the population the organisations needed to attract before their influence was visible in the whole population was larger in urban areas than in rural areas. As the population density increases, the number of possible social interactions between people increases. Within urban areas there was a wider range of social organisations and networks, which weakened the influence of any one organisation. As Gamm and Putnam (1999) have hypothesised, the reverse mechanism should be in effect in sparsely populated areas, and it is easy to see that the number and strength of the connections between the members would be larger in smaller communities. Thus, the free church could more easily shape people’s interests and perceptions in rural areas.

## Discussion

Social networks have been shown to influence reproductive behaviours in post-transitional societies (Balbo and Barban 2014; Keim 2011; Rossier and Bernardi 2009). However, the role of changing social relations has not received as much attention in the study of the historical European fertility decline of the late nineteenth and early twentieth-centuries. This study shows that the adoption of fertility limitation during the transition was associated with the creation and diffusion of ideational effects within large social network organisations. Furthermore, by applying a social network perspective, the results show that the strength of the effect was dependent on the structure of the social networks in terms of size, density, and homogeneity.

In the Swedish context, the emergence of voluntary association mass movements in the late nineteenth century represented a radical shift in the landscape of social relations (Ambjörnsson 1998; Horgby 1993; Lundkvist 1980). By utilising unique Swedish data on the size of local voluntary association groups (Andrae and Lundkvist 1998), we have estimated the sizes of the free churches, temperance associations, and workers’ organisations at the parish level. By combining these estimates with census data from 1890 and 1900 for individual-level indicators of fertility, this study has assessed the impact of voluntary association size on individual-level net fertility during the demographic transition. As expected, the results show that the larger voluntary associations were at the parish level the lower the marital net fertility was within the parish. These observations hold true for the free churches and the workers’ organisations when estimating the effect of voluntary associations on fertility through mixed-effects Poisson regression models that control for adjustment factors and geographical heterogeneity. This shows that there was an association between social network organisation size and structure at the aggregate level and between social network organisation size and couples’ reproductive practices at the individual level.

While the growth of the Swedish voluntary associations facilitated social change, the different movements had quite different effects on net fertility. In 1890 the free churches were the only voluntary association that displayed a clear negative relationship to net fertility, and net fertility was 5% lower within parishes with strong free churches than in parishes with no free churches. The relationship strengthened in 1900 to almost 10%, and at this point the effect of workers’ organisations on net fertility was also negative. The workers’ movement comprised quite homogenous networks because they consisted primarily of young men from the working classes, which strengthened the mechanisms. Although the free churches were not as socially homogenous, they created dense connections between their members through strong social control. The importance of the structure of the organisations for the effect of social network mechanisms is highlighted by the temperance organisations, which did not show any relationship to net fertility. This was likely because, although they were large associations and had morality and restraint central to their agenda, they were not as dense as the other groups because the connections between nodes were sparse and weak due to high membership turnover (Lundkvist 1977).

In addition, the results question the importance of social homogeneity of networks because the effects on fertility were stronger for the socially heterogeneous free churches than the more homogenous temperance associations. However, this could indicate differences between types of diffusion processes. While the probability of joining an association was higher in homogenous networks during the time period studied here (Sandell and Stern 1998), once they joined each member represented a link to other networks outside the association. A socially diverse membership would thereby increase the number of possible nodes of contact between the members and the rest of society, thus increasing the probability of diffusion of ideational factors to the larger population. Watkins and Danzi (1995) have shown the strength of weak ties for the diffusion of innovation in heterogeneous networks because such links are more likely to span across social boundaries and thus make new ideas more likely to take hold in these networks, thus facilitating social change. These findings are in line with the results of this study.

The probability of joining a voluntary association was not only affected by the structure of the network, but also by the context in which these social relations were embedded (Sandell and Stern 1998). Similarly, the results show that not only the structure of the network, but also the context of the networks, were important for how voluntary association size affected net fertility. By introducing an interaction between voluntary association size and population density, the results show that the negative effect of free church size was most distinct in rural areas. While the size of workers’ organisations showed no relation to net fertility in rural areas, the effect in urban areas was strong, showing differences in predicted net fertility of 7%.

The connection between respectability and the adoption of birth control practices within voluntary associations found in qualitative studies is corroborated by the results in this article (Ambjörnsson 1998; Horgby 1993; Kling 2007). To be a conscientious and respectable member of a voluntary association at the turn of the century entailed high levels of sexual morality and restraint, resulting in couples having smaller families. This effect, in turn, had macro-level consequences, and depending on the context and structure of the organisations, voluntary associations’ influence at the parish level affected individual-level fertility and shaped the pace of the fertility decline in Sweden. This is in line with the literature that argues that couples’ reproductive practices were dependent on how gendered social and cultural identities were formed within social networks (Junkka and Edvinsson 2016; Praz 2007; Szreter 1996; Warg 2002). However, this process was accompanied by other large economic and social structural changes.

Structural changes, such as mortality decline or economic modernisation, disrupted the pre-transitional demographic regime, creating new circumstances for family formation. How individuals acted in response to this depended on how they could incorporate new reproductive practices into their lives. Social class has been shown to have an impact on the pace of the transition by creating fertility differentials (Dribe and Scalone 2014; Dribe et al. 2014), and such results are corroborated by the results in this study. What has not been shown before is that social network mechanisms were important for the process during the transition in the same way as in post-transitional societies (Balbo and Barban 2014; Bernardi and Klaerner 2014; Keim 2011; Rossier and Bernardi 2009). Individual members of voluntary associations observed, learned from their peers, and adapted their actions to the social pressures found within the networks in which they were embedded. In this sense, voluntary association organisations facilitated new ways of incorporating reproductive practices into perceptions of family life.

The choice of data involved a trade-off which introduced some important limitations. It is not possible to draw conclusions on causal links or the direction of causality from aggregate-level voluntary association size. Therefore, the results could be an effect of reverse causation, and it is easy to assume that having fewer children would make it easier, especially for women, to join an association. Another limitation is the possibility of confounding factors not controlled for in the regression model causing both the size of voluntary associations and fertility decline. Although the literature is not in agreement, several studies link voluntary association growth to industrialisation (Anderson 1971; Curtis et al. 1992; Gamm and Putnam 1999; Gorsky 1998; Lundkvist 1980), which in turn is also associated with fertility decline (Dribe 2009; Notestein 1953). Even though the regression model presented here tries to control for industrialisation through proxy variables, the possibility still remains that industrialisation is the underlying cause. Another potential confounding factor is the existence of social groups acting as forerunners driving the diffusion of birth control and the diffusion of association affiliation (Van Bavel 2004; Vanhaute and Matthys 2007). Without individual-level data on both the timing of joining an association and the timing of births, the causal link cannot be determined. However, these effects have been investigated in both qualitative studies and quantitative longitudinal studies within confined areas (Horgby 1993; Junkka and Edvinsson 2016; Kling 2007; Warg 2002), and this study builds on these previous findings by testing if the observations can be generalised across a whole nation. The results thereby contribute to the literature by exploring the generalisability of the hypothesis at the cost of determining causality.

In conclusion, the results of this study show that the use of social network theory in the study of fertility change helps us to include norms and values in the explanation for such change by shifting the focus from individual attributes to practices in the context of social relations. The theory helps us to shift the theoretical focus from the innovation of birth control to the meaning and perception of birth control and thereby to assert the importance of the context in which human action is embedded. However, because this study lacks individual-level information on voluntary association connections and instead relies on aggregate-level information, the causal link between these social relations and changing reproductive practices cannot be determined and needs to be studied further. The urban and rural differences also suggest that further research using spatial analysis could uncover even more distinct geographical patterns. The scope of this article has moreover been limited to the beginning of the fertility transition, and studies on the following decades might reveal a different pattern, especially considering the growth of the political organisations and trade unions during the first decades of the twentieth century.

## Appendix

The datasets used in this analysis are available at the Swedish National Data Service with the identifier https://doi.org/10.5878/000124 and at the North Atlantic Population Project with the identifier https://doi.org/10.18128/D040.V2.2. The code to reproduce the analysis is available at GitHub, https://github.com/junkka/voluntary-associations.

**Table 5:** Interaction effects of voluntary association size and census year on the number of children under 5 years of age in 1890 and 1900 in Sweden shown as incident rate ratios (IRR) and standard errors (SE) from the Poisson regression. Source: Minnesota Population Center (2015) and Andrae and Lundkvist (1998)

	Variable	IRR	SE	*P* value
*Intercept*		1.41	0.006	0.00
Free church	None (Ref.)	1.00		
	Small	0.97	0.006	0.00
	Medium	0.96	0.006	0.00
	Large	0.94	0.007	0.00
Temperance association	None (Ref.)	1.00		
	Small	1.01	0.005	0.16
	Medium	0.99	0.006	0.37
	Large	0.99	0.007	0.25
Workers’ movement	None (Ref.)	1.00		
	Small	1.00	0.008	0.58
	Medium	1.03	0.009	0.00
	Large	1.06	0.024	0.02
Wife’s age	20–24	0.77	0.004	0.00
	25–29 (Ref.)	1.00		
	30–34	1.00	0.003	0.13
	34–39	0.87	0.003	0.00
	40–44	0.61	0.003	0.00
	45–49	0.21	0.005	0.00
Wife’s occupation	Not in workforce (Ref.)	1.00		
	In workforce	0.70	0.012	0.00
Husband’s HISCLASS	Skilled workers (Ref.)	1.00		
	Elite	0.82	0.009	0.00
	Lower middle class	0.93	0.004	0.00
	Farmer	1.07	0.003	0.00
	Unknown	0.87	0.004	0.00
	Unskilled workers	1.04	0.003	0.00
Teachers per child	< 0.019 (Ref.)	1.00		
	$\ge$ 0.019–< 0.027	0.98	0.004	0.00
	$\ge$ 0.027–< 0.038	0.97	0.004	0.00
	$\ge$ 0.038–< 0.500	0.94	0.005	0.00
Infant mortality rate		0.89	0.011	0.00
Urbanisation	Rural (Ref.)	1.00		
	Urban	0.94	0.010	0.00
Year	1890 (Ref.)	1.00		
	1900	1.02	0.006	0.00
Free church * Year	None * Year (Ref.)	1.00		
	Small * Year	0.99	0.007	0.13
	Medium * Year	0.96	0.007	0.00
	Large * Year	0.96	0.007	0.00
Temperance * Year	None * Year (Ref.)	1.00		
	Small * Year	0.98	0.008	0.03
	Medium * Year	0.98	0.009	0.01
	Large * Year	1.00	0.009	0.79
Workers * Year	None * Year (Ref.)	1.00		
	Small * Year	1.01	0.009	0.38
	Medium * Year	0.96	0.011	0.00
	Large * Year	0.89	0.023	0.00
Summary	Observations		1,068,224	
	Random effect groups		2521	
	Degrees of freedom		37	
	Standard deviation of			
	parish random effects		0.101	
	Deviance residuals		961,513	
	Residual degrees of freedom		1,068,187	
	AIC		2,519,926

**Table 6:** Interaction effects of voluntary association size and urbanisation on the number of children under 5 years of age in 1890 and 1900 in Sweden shown as incident rate ratios (IRR) and standard errors (SE) from the Poisson regression. Source: Minnesota Population Center (2015) and Andrae and Lundkvist (1998)

	Variable	IRR	SE	*P* value
*Intercept*		1.42	0.006	0.00
Free church	None (Ref.)	1.00		
	Small	0.97	0.005	0.00
	Medium	0.95	0.006	0.00
	Large	0.93	0.006	0.00
Temperance association	None (Ref.)	1.00		
	Small	1.00	0.004	0.98
	Medium	1.00	0.005	0.95
	Large	0.99	0.006	0.15
Workers’ movement	None (Ref.)	1.00		
	Small	1.00	0.005	0.66
	Medium	1.00	0.007	0.95
	Large	0.98	0.009	0.03
Wife’s age	20–24	0.77	0.004	0.00
	25–29 (Ref.)	1.00		
	30–34	1.00	0.003	0.13
	34–39	0.87	0.003	0.00
	40-44	0.61	0.003	0.00
	45–49	0.21	0.005	0.00
Wife’s occupation	Not in workforce (Ref.)	1.00		
	In workforce	0.70	0.012	0.00
Husband’s HISCLASS	Skilled workers (Ref.)	1.00		
	Elite	0.82	0.009	0.00
	Lower middle class	0.93	0.004	0.00
	Farmer	1.07	0.003	0.00
	Unknown	0.87	0.004	0.00
	Unskilled workers	1.04	0.003	0.00
Teachers per 100 child	< 0.019 (Ref.)	1.00		
	$\ge$ 0.019–< 0.027	0.98	0.004	0.00
	$\ge$ 0.027–< 0.038	0.97	0.004	0.00
	$\ge$ 0.038–< 0.500	0.94	0.006	0.00
Infant mortality rate		0.89	0.011	0.00
Urbanisation	Rural (Ref.)	1.00		
	Urban	0.94	0.032	0.07
Year	1890 (Ref.)	1.00		
	1900	0.99	0.003	0.00
Free church * Urban	Zero * Urban (Ref.)	1.00		
	Small * Urban	1.02	0.023	0.32
	Medium * Urban	1.02	0.024	0.37
	Large * Urban	1.00	0.025	0.97
Temperance * Urban	Zero * Urban (Ref.)	1.00		
	Small * Urban	1.06	0.030	0.06
	Medium * Urban	0.98	0.030	0.63
	Large * Urban	1.00	0.032	0.98
Workers * Urban	Zero * Urban (Ref.)	1.00		
	Small * Urban	0.99	0.013	0.41
	Medium * Urban	0.99	0.014	0.62
	Large * Urban	0.92	0.017	0.00
Summary	Observations		1,068,224	
	Random effect groups		2521	
	Degrees of freedom		37	
	Standard deviation of			
	parish random effects		0.101	
	Deviance residuals		961,451	
	Residual degrees of freedom		1,068,187	
	AIC		2,519,879	
